# Prognostic Factors Associated with Ocriplasmin Efficacy for the Treatment of Symptomatic Vitreomacular Adhesion and Full-thickness Macular Hole: Analysis from Four Studies

**DOI:** 10.18502/jovr.v16i1.8250

**Published:** 2021-01-20

**Authors:** Brian C. Joondeph, Paul Willems, Thomas Raber, Luc Duchateau, Joseph Markoff

**Affiliations:** ^1^Colorado Retina Associates, Denver, Colorado, USA; ^2^Oxurion, Leuven, Belgium; ^3^Biometrics Research Group, Ghent University, Gent, Belgium; ^4^Wills Eye Hospital, Philadelphia, Pennsylvania, USA; ^5^Thomas Jefferson Medical College, Philadelphia, Pennsylvania, USA

**Keywords:** Ocriplasmin, Full-thickness Macular Hole, Vitreomacular Adhesion, Symptomatic Vitreomacular Adhesion, Vitreomacular Traction, Vitreoretinal Interface

## Abstract

**Purpose:**

To assess the effect of patient baseline characteristics on the efficacy of ocriplasmin treatment for symptomatic vitreomacular adhesion (VMA) with full-thickness macular hole (FTMH) from phase 3/4 studies.

**Methods:**

Patients with symptomatic VMA and FTMH at baseline and receiving ocriplasmin treatment 125 μg were pooled from the MIVI-TRUST, OASIS, and ORBIT studies. Multivariable logistic regression analysis was used to evaluate whether patient baseline characteristics were predictors of having VMA resolution by Day 28 and FTMH closure by Month 6.

**Results:**

Two hundred and seventy-four patients receiving ocriplasmin treatment were assessed. Overall, 22.6% (62/274) of the patients experienced both VMA resolution by Day 28 and non-surgical FTMH closure by Month 6. Patients with FTMH ≤250 µm at baseline had a significantly higher success rate compared to those with FTMH >400 µm (29.9% [41/137] vs 2.2% [1/48]; *P* = 0.009). In patients with VMA resolution by Day 28, both small FTMH size (*P* = 0.001) and FTMH width at RPE (*P* = 0.012) were significantly associated with a higher FTMH closure rate. Patients with VMA resolution had higher rates of FTMH closure. Previously identified baseline predictive factors, including age, lens status, or presence of epiretinal membrane (ERM) were not found to be predictive of both VMA release and FTMH closure.

**Conclusion:**

The analysis revealed that FMTH ≤250 µm was the only factor predictive for achieving both pharmacological VMA resolution by Day 28 and nonsurgical FTMH closure by Month 6; neither lens status or presence of ERM, previously identified baseline characteristics favoring VMA resolution, showed statistically significant predictive power for both outcomes.

##  INTRODUCTION

Aging of the eye often leads to separation between the posterior vitreous cortex and the internal limiting membrane, known as posterior vitreous detachment (PVD).^[1,2]^ This process may be affected by vitreomacular adhesion (VMA), or adherence of the vitreous cortex to the macula after partial detachment.^[3,4,5]^ Symptomatic VMA (also referred to as vitreomacular traction) can occur if mechanical forces are large enough to cause anatomical changes to the macula.^[6,7]^ Effects resulting from symptomatic VMA may also lead to the development of a full-thickness macular hole (FTMH).^[4]^ The occurrence of VMA and FTMH can lead to visual disturbances such as decreased visual acuity, photopsia, metamorphopsia, scotomas, and may result in irreversible vision loss if left untreated.^[3,4,8,9,10,11,12]^

Treatment options for symptomatic VMA include watchful waiting, vitrectomy, pneumatic vitreolysis, and pharmacological vitreolysis with ocriplasmin. Ocriplasmin was approved in the US in 2012 and the EU in 2013 based on the results of two pivotal phase 3 clinical trials (MIVI-TRUST) that established its efficacy and safety in patients with symptomatic VMA with or without an associated FTMH ≤400 μm.^[13]^ An earlier post hoc analysis of the pivotal trials suggested that the efficacy of ocriplasmin may be increased by patient baseline characteristics, including younger age, phakic lens status, focal VMA, absence of epiretinal membrane (ERM), and presence of FTMH.^[14]^ Subsequently, both prospective and retrospective studies ranging from 5 to 74 eyes were undertaken that assessed the effect of these baseline factors with respect to VMA release.^[13,15,16,17,18,19,20,21,22,23,24,25,26,27,28,29,30,31,32]^ VMA release rates in these studies ranged from 0% to 71%, with 14 of 18 studies showing higher efficacy than the pivotal phase 3 trial rate of 26.5% VMA release at Day 28.^[13]^ A meta-analysis of these studies, which also included the phase 3 pivotal trials, confirmed that focal VMA, absence of ERM, phakic lens status, and younger age were all positive predictive factors for VMA release.^[33]^

The rate of FTMH closure for ocriplasmin-treated eyes was 40.6% in the pivotal clinical trials and 30.0% in the OASIS study.^[13,34]^ Although analysis of baseline predictive factors has resulted in real-world rates of VMA release higher than those in the pivotal phase 3 trials, multiple real-world studies have reported FTMH closure rates lower than those observed in these studies, suggesting that the predictive factors for FTMH closure may not be the same as those for VMA release and are not as well understood.^[24,28,35]^ For instance, the absence of ERM did not have a clear association with FTMH closure in the MIVI-TRUST trials.^[36]^ In addition, the predictive value of successful VMA release on FTMH closure remains unclear; there was no clear association between VMA release and FTMH closure in the MIVI-TRUST trials,^[36]^ although a recent study showed a strong association between VMA release and FTMH closure.^[37]^

Although the baseline factors associated with VMA resolution and FTMH closure have been investigated individually, to our knowledge no study has assessed factors that may predict both VMA resolution and FTMH closure following ocriplasmin treatment. The current study aimed at assessing the baseline factors that may be predictive of both VMA release together with FTMH closure in patients treated with ocriplasmin in the completed phase 3/4 studies.

##  METHODS

### Study Population

Patients diagnosed with both symptomatic VMA and FTMH at baseline and receiving treatment of ocriplasmin 125 μg were pooled from the MIVI-TRUST, OASIS, and ORBIT studies. MIVI-TRUST (NCT00781859 and NCT00798317) consisted of two phase 3, prospective, randomized, multicenter, double-blind, placebo-controlled clinical trials (TG-MV-006 and TG-MV-007) in which patients were randomized to receive a single intravitreal ocriplasmin (125 μg) or placebo injection.^[13]^ OASIS (NCT01429441) was a phase 3b, randomized, multicenter, double-masked, sham-controlled clinical trial in which patients were randomized to receive a single intravitreal ocriplasmin 125 μg injection or sham treatment.^[34]^ ORBIT (NCT02079883) was a phase 4, prospective, multicenter, observational study to assess a single intravitreal ocriplasmin injection of 125 μg.^[38]^ Full details of individual study designs, treatment plans, and adherence to ethics practices have been published elsewhere.^[13,34][38]^

### Baseline Demographics and Patient Characteristics

The following baseline demographic and ocular characteristics were evaluated in the study population based on availability across datasets: age (<65 years, ≥65 years), lens status (phakic, pseudophakic), ERM status (present, absent), ellipsoid zone (EZ) status (normal, abnormal), subretinal fluid (SRF) status (present, absent), BCVA (<65, 65–75, >75 ETDRS letters), diameter of VMA (≤1500 μm, >1500 μm), width of FTMH (≤250, >250–400, >400 μm), and width of FTMH at the retinal pigment epithelium (RPE) (≤600 μm, >600 μm) (Supporting Information Table S1).

**Table 1:** Patient demographics and ocular baseline characteristics in the four studies and the integrated dataset


**Characteristic**	MIVI-TRUST*( ***N*** ** = 106)**	**OASIS (** ***N*** ** = 50)**	**ORBIT(** ***N*** ** = 118)**	**Integrated(** ***N*** ** = 274)**
Age (years)				
Mean (SD)	68.7 (7.4)	66.5 (6.3)	66.7 (7.3)	67.5 (7.2)
Median	69.0	65.5	66.0	67.0
Min, Max	48, 85	49, 79	45, 88	45, 88
Age group (years), *n* (%)				
<65 years	31 (29.2)	20 (40.0)	42 (35.6)	93 (33.9)
≥65 years	75 (70.8)	30 (60.0)	76 (64.4)	181 (66.1)
Sex, *n* (%)		
Male	22 (20.8)	10 (20.0)	28 (23.7)	60 (21.9)
Female	84 (79.2)	40 (80.0)	90 (76.3)	214 (78.1)
Race, *n* (%)				
White	99 (93.4)	46 (92.0)	105 (89.0)	250 (91.3)
Black or African American	3 (2.8)	4 (8.0)	9 (7.6)	16 (5.8)
Asian	2 (1.9)	0 (0)	3 (2.5)	5 (1.8)
Other	2 (1.9)	0 (0)	1 (0.9)	3 (1.1)
Lens status, *n* (%)				
Phakic	81 (76.4)	43 (86.0)	93 (78.8)	217 (79.2)
Pseudophakic	25 (23.6)	7 (14.0)	24 (20.3)	56 (20.4)
Aphakic	0 (0)	0 (0)	1 (0.9)	1 (0.4)
ERM status, *n* (%)				
Present	18 (17.0)	6 (12.0)	14 (11.9)	38 (13.9)
Absent	82 (77.3)	44 (88.0)	104 (88.1)	230 (83.9)
Missing	6 (5.7)	0 (0)	0 (0)	6 (2.2)
EZ status, *n* (%)				
Abnormal	0 (0)	49 (98.0)	116 (98.3)	165 (60.2)
Normal	0 (0)	1 (2.0)	2 (1.7)	3 (1.1)
Missing	106 (100)	0 (0)	0 (0)	106 (38.7)
SRF status, *n* (%)				
Present	77 (72.7)	49 (98.0)	0 (0)	126 (46.0)
Absent	26 (24.5)	1 (2.0)	118 (100)	145 (52.9)
Missing	3 (2.8)	0 (0)	0 (0)	3 (1.1)
BCVA (ETDRS letters), *n* (%)				
<65	89 (84.0)	37 (74.0)	96 (81.4)	222 (81.0)
65–75	16 (15.1)	12 (24.0)	19 (16.1)	47 (17.2)
>75	1 (0.9)	1 (2.0)	3 (2.5)	5 (1.8)
FTMH size, *n* (%)				
≤250 μm	48 (45.3)	23 (46.0)	66 (55.9)	137 (50.0)
>250–400 μm	38 (35.9)	17 (34.0)	33 (28.0)	88 (32.1)
>400 μm	19 (17.9)	10 (20.0)	19 (16.1)	48 (17.5)
Missing	1 (0.9)	0 (0)	0 (0)	1 (0.4)
VMA diameter, *n* (%)				
≤1500 μm	90 (84.9)	43 (86.0)	110 (93.2)	243 (88.7)
>1500 μm	3 (2.8)	2 (4.0)	1 (0.9)	6 (2.2)
Missing	13 (12.3)	5 (10.0)	7 (5.9)	25 (9.1)
FTMH width at RPE (μm)				
*n*	104	50	0	154
Mean (SD)	647.1 (283.8)	634.2 (320.8)	–	642.9 (295.4)
Median	611.0	596.0	–	611.0
Min, Max	113, 1572	164, 2120	–	113, 2120
FTMH width at RPE, *n* (%)				
≤600 μm	49 (46.2)	25 (50.0)	0 (0)	74 (27.0)
>600 μm	55 (51.9)	25 (50.0)	0 (0)	80 (29.2)
Missing	2 (1.9)	0 (0)	118 (100)	120 (43.8)
${}^{*}$MIVI-TRUST consisted of two phase 3 clinical trials (NCT00781859 and NCT00798317) BCVA, best-corrected visual acuity; ERM, epiretinal membrane; ETDRS, Early Treatment Diabetic Retinopathy Study; EZ, ellipsoid zone; FTMH, full-thickness macular hole; RPE, retinal pigment epithelium; SD, standard deviation; SRF, subretinal fluid; VMA, vitreomacular traction				

**Table 2:** Rates of VMA resolution and FTMH closure in the four studies and the integrated dataset


	MIVI-TRUST* **** ***n*** ** (%)**	**OASIS** **** ***n*** ** (%)**	**ORBIT** **** ***n*** ** (%)**	**Integrated** **** ***n*** ** (%)**
Number of patients	106	50	118	274
VMA resolution	53 (50.0)	27 (54.0)	74 (62.7)	154 (56.2)
FTMH closure	43 (40.6)	15 (30.0)	38 (32.2)	96 (35.0)
VMA resolution: Yes FTMH closure: Yes	24 (22.6)	8 (16.0)	30 (32.2)	62 (22.6)
VMA resolution: Yes FTMH closure: No					29 (27.4)	19 (38.0)	44 (37.3)	92 (33.6)
VMA resolution: No FTMH closure: Yes	19 (17.9)	7 (14.3)	8 (6.8)	34 (12.4)				
VMA resolution: No FTMH closure: No					24 (22.6)	8 (16.0)	30 (25.4)	86 (31.4)
*MIVI-TRUST consisted of two phase 3 clinical trials (NCT00781859 and NCT00798317) FTMH, full-thickness macular hole; VMA, vitreomacular adhesion								

**Table 3:** Univariable logistic regression analysis for the effect of patient demographics and ocular baseline characteristics on VMA resolution by Day 28 and FTMH closure by Month 6 in the integrated dataset


	**VMA resolution**		**FTMH closure**		**VMA resolution + FTMH closure**	
**Characteristic**	**Status**	**Success (%)**	**** ***P-*** **value**	**Success (%)**	**** ***P*** **-value**	**Success (%)**	**** ***P*** **-value**
Age	<65 years	65/93 (69.9)	0.0015	32/93 (34.4)	0.9783	22/93 (23.7)	0.735
	≥65 years	89/181 (49.2)		64/181 (35.4)		40/181 (22.1)		
Lens Status	Phakic	130/217 (59.9)	0.0129	71/217 (32.7)	0.1888	47/217 (21.7)	0.647
	Pseudophakic	23/56 (41.1)		24/56 (42.9)		14/56 (25.0)		
ERM status	Present	12/38 (31.6)	0.0028	13/38 (34.2)	0.7999	4/38 (10.5)	0.067
	Absent	137/230 (59.6)		81/230 (35.2)		56/230 (24.3)		
EZ status	Normal	1/3 (33.3)	0.3667	3/3 (100)	0.9852	1/3 (33.3)	0.645
	Abnormal	100/165 (60.6)		50/165 (30.3)		37/165 (22.4)		
SRF status	Present	67/126 (53.2)	0.2874	48/126 (38.1)	0.2327	28/126 (22.2)	0.124
	Absent	85/145 (58.6)		46/145 (31.7)		33/145 (22.8)		
BCVA (ETDRS letters)	<65	127/222 (57.2)	0.6215	71/222 (32.0)	0.0606	49/222 (22.1)	0.645
	65–75	25/47 (53.2)		22/47 (46.8)		13/47 (27.7)		
	>75	2/5 (40.0)		3/5 (60)		0/5 (0)		
VMA diameter	≤1500 µm	144/243 (59.3)	0.7324	87/243 (35.8)	0.8514	56/243 (23.0)	0.489
	>1500 µm	3/6 (50)		2/6 (33.3)		2/6 (33.3)		
FTMH size	≤250 µm	75/137 (54.7)	0.3412	67/137 (48.9)	<0.0001	41/137 (29.9)	0.009
	>250–400 µm	54/88 (61.4)		26/88 (29.6)		19/88 (21.6)		
	>400 µm	24/48 (50)		2/48 (4.2)		1/48 (2.2)		
FTMH width at RPE	≤600 µm	40/74 (54.1)	0.5185	38/74 (51.4)	0.0004	21/74 (28.4)	0.015
	>600 µm	39/80 (48.8)		19/80 (23.8)		10/80 (12.5)		
BCVA, best-corrected visual acuity; ERM, epiretinal membrane; ETDRS, Early Treatment Diabetic Retinopathy Study; EZ, ellipsoid zone; FTMH, full-thickness macular hole; RPE, retinal pigment epithelium; SRF, subretinal fluid; VMA, vitreomacular adhesion							

**Table 4:** univariable logistic regression analysis for the effect of patient demographics and ocular baseline characteristics on FTMH closure by Month 6 for patients with VMA resolution by Day 28 in the integrated dataset


**Patient Characteristic**	**Status**	**Success (%)**	**** ***P*** **-value**
Age	<65 years	22/65 (33.8)	0.177
	≥65 years	49/89 (55.1)		
Lens Status	Phakic	47/130 (36.2)	0.027
	Pseudophakic	14/23 (60.9)		
ERM status	Present	4/12 (33.3)	0.619
	Absent	56/137 (40.9)		
EZ status	Normal	1/1 (100.0)	0.986
	Abnormal	37/100 (37.0)		
SRF status	Present	28/67 (41.8)	0.231
	Absent	33/85 (38.8)		
BCVA (ETDRS letters)	<65	49/127 (38.6)	0.434
	65–75	13/25 (52.0)		
	>75	2/2 (100.0)		
VMA diameter	≤1500 µm	56/144 (38.9)	0.311
	>1500 µm	2/3 (66.7)		
FTMH size	≤250 µm	41/75 (54.7)	0.001
	>250–400 µm	19/54 (35.2)		
	>400 µm	1/24 (4.2)		
FTMH Width at RPE	≤600 µm	21/40 (52.5)	0.012
	>600 µm	10/39 (25.6)		
BCVA, best-corrected visual acuity; ERM, epiretinal membrane; ETDRS, Early Treatment Diabetic Retinopathy Study; EZ, ellipsoid zone; FTMH, full-thickness macular hole; RPE, retinal pigment epithelium; SRF, subretinal fluid; VMA, vitreomacular adhesion			

**Supplemental Table 1:** Availability of baseline characteristics and outcome measures in the ocriplasmin studies

	**MIVI-TRUST**	**OASIS**	**ORBIT**
**Outcome measures**			
Pharmacological VMA resolution at Day 28, post-resolution vitrectomy considered as a failure	Yes	Yes	No
Pharmacological VMA resolution at Day 28, post-resolution vitrectomy not considered as a failure	Yes	Yes	Yes
Non-surgical FTMH closure by end of study (post-closure vitrectomy not considered as a failure)	EOS (up to M6)	M6 EOS (up to M24)	M6 M12
**Baseline characteristics**			
Age (Years)	Available	Available	Available
Lens status	Phakic Pseudophakic	Phakic Pseudophakic	Phakic Pseudophakic Aphakic
ERM	Present Absent	Present Minimal Present Significant Absent	Present* Absent*
EZ	Not available	Definitely Fully Intact Likely site(s) of Incomplete EZ Definite site(s) of Incomplete EZ Unable to grade	Normal* Abnormal*
SRF	Present Absent	Present Absent	Present* Absent*
BCVA (ETDRS)	Available	Available	Available after transformation
FTMH size (μm)	Available	Available	Available
VMA diameter	Available	Available	Available
FTMH width at RPE	Available	Available	Not available
*Assessed by SD-OCT BCVA, best-corrected visual acuity; ELM, external limiting membrane; EOS, end of study; ERM, epiretinal membrane; ETDRS, Early Treatment Diabetic Retinopathy Study; EZ, ellipsoid zone; FTMH, full-thickness macular hole; M, month; RPE, retinal pigment epithelium; SD-OCT, spectral-domain optical coherence tomography; SRF, subretinal fluid; VMA, vitreomacular adhesion			

For the MIVI-TRUST trials, the presence and size of VMA and FTMH status at baseline were assessed by a central reading center (CRC), using mandatory time-domain optical coherence tomography (TD-OCT) as required per protocol; additional spectral-domain (SD)-OCT readings if available were only used as supportive information for evaluation of individual cases.^[13]^ FTMH was defined as a macular hole with bare/exposed RPE, with the largest of the minimum hole width measurements considered as the hole width based on macular thickness map (MTM) or fast macular thickness map (FMTM) scans. In the more recent OASIS study, the presence and size of VMA and FTMH status at baseline were assessed by a CRC using SD-OCT.^[34]^ FTMH diameter was defined as the largest of the minimum hole width measurement. Although patients were enrolled in the OASIS trial based on favorable baseline characteristics,^[14]^ determination of ocular characteristics differed between investigator and CRC assessment, resulting in inclusion of some patients despite their CRC assessment meeting exclusion criteria in retrospect (FTMH > 400 μm, presence or ERM).^[34]^ In the ORBIT study, the presence of VMA and FTMH was determined by SD-OCT according to the treating physician before enrollment and reviewed independently by a CRC in retrospect. FTMH diameter was defined as the greatest width of the minimum distance between sides of the FTMH measured within the middle two thirds of the retina (not at surface and not at RPE) in any line of the 49-line volume scan. The review of the presence of VMA and FTMH by the CRC was performed post-treatment in all studies and was not used for treatment decisions.

EZ status was evaluated in the central macular region in all studies. SRF assessments were defined in each of the studies. In the MIVI-TRUST trials, SRF was a measure of the fluid beneath retina to other material perpendicular to Bruch's membrane at the foveal center from the retina to the RPE, not including fluid within the retinal layer (cysts) or fluid below the RPE. In the OASIS study, three foveal center point measurements were taken, including SRF, RPE elevation and/or subretinal hyper-reflective material (SHRM) such as choroidal neovascularization, and total retinal thickness. The total retinal thickness measurement included the RPE layer, RPE elevation, any SHRM, any SRF, and the retina at the foveal center. When a value was not reported for SRF or RPE elevation and/or SHRM, it was considered not present or ungradable. In the ORBIT study, SRF was considered present if it was identified in any line scan in the absence of FTMH.

### Statistical Analysis

The integrated database included all patients who presented with symptomatic VMA and FTMH at baseline, were treated with ocriplasmin 125 μg, and had both a baseline assessment and at least one follow-up visit. Three different variables (i.e., treatment response) were considered: pharmacological resolution of VMA by Day 28 (VMAres), nonsurgical FTMH closure by Month 6 (MHclos), and combined success when experiencing both events (VMAres + MHclos). First, the effect of each patient baseline characteristic on success was evaluated separately in a univariable logistic regression model that also included study as a fixed-effects factor to accommodate for the clustering in the data due to combining data from different studies. Next, all patient baseline characteristics that were significant at the 5% significance level were included in a multivariable regression analysis to identify independent patient baseline characteristics that were significantly associated with treatment success. Additionally, the same analysis was performed for MHclos for those patients that experienced VMAres.

##  RESULTS

### Demographics and Baseline Characteristics

A total of 274 patients were pooled from the MIVI-TRUST, OASIS, and ORBIT studies on the basis of having both symptomatic VMA and FTMH at baseline and having received a single intravitreal injection of ocriplasmin 125 μg. Demographics and ocular characteristics are shown in Table 1. Overall, the demographics and ocular characteristics were generally comparable in patients across the three datasets. The mean age of the patients was 67.5 years, with an age range of 45–88 years. Seventy-nine percent of the patients had phakic lens status. A majority (60.2%) of patients had EZ status categorized as abnormal, and 46.0% of patients had SRF present (Table 1).

### VMA Resolution

An average of 56.2% (154/274) of eligible patients experienced VMA resolution by Day 28 (Table 2). The proportion of patients experiencing VMA resolution by Day 28 in this patient subpopulation with FTMH at baseline was consistently higher than or equal to 50% for all studies (Table 2).

VMA resolution by Day 28 was achieved significantly more frequently in younger patients, in the absence of ERM at baseline and for eyes with phakic lens status at baseline (Table 3). In the multivariable model including these three variables, age (*P *= 0.006) and ERM status at baseline (*P *= 0.010) remained significant, but not lens status at baseline (*P *= 0.179).

### FTMH Closure

The average rate of FTMH closure by Month 6 in the integrated dataset was 35.0% (96/274) (Table 2). Closure rates varied from 30.0% for the OASIS database and 32.2% for the ORBIT study to 40.6% for the MIVI-TRUST trials (Table 2). FTMH closure by Month 6 occurred significantly more often with smaller FTMH size and smaller FTMH width at RPE (Table 3). We did not construct the multivariable model as these two variables are highly interrelated: the percentage of patients with FTMH width at RPE ≤600 µm decreases from 64.8% (46/71) to 45.5% (25/55) and 10.7% (3/28) for the ≤250 µm, >250–400 µm, and >400 µm FTMH size categories, respectively.

VMA resolution by Day 28 was a positive predictor for FTMH closure by Month 6. Patients with VMA resolution by Day 28 had a higher percentage of MH closure of 40.3% (62/154) compared to patients without VMA release equal to 28.3% (34/120) (*P *= 0.028). Within the group of patients who had VMA resolution by Day 28, MH closure by Month 6 occurred significantly more for eyes with pseudophakic lens status at baseline, with smaller FTMH size and smaller FTMH width at RPE (Table 4). In the multivariable models including lens status with one of the two FTMH measurements at a time, lens status was no longer significant (*P *= 0.244 with FTMH size and *P *= 0.173 with FTMH width at RPE), nor was the FTMH size (*P *= 0.057), but the FTMH width at RPE remained significant (*P* < 0.001).

### VMA Resolution and FTMH Closure

Overall, 22.6% (62/274) of patients in this analysis experienced both VMA resolution by Day 28 and FTMH closure by Month 6 (Table 2). In contrast, 12.4% (34/274) experienced FTMH closure by Month 6 without VMA resolution by Day 28; 33.6% (92/274) experienced VMA resolution by Day 28 without FTMH closure by Month 6; and 31.4% (86/274) showed neither VMA resolution by Day 28 nor non-surgical FTMH closure by Month 6 (Table 2).

Univariable logistic regression analysis revealed a statistically significant effect for FTMH size at baseline on treatment success (*P *= 0.009; Table 3), with success increasing from 2.2% for patients with FTMH size at baseline >400 µm to 21.6% for patients with FTMH size at baseline between 250 and 400 µm, and further to 29.9% for patients with FTMH size at baseline <250 µm. Similarly, FTMH width at RPE at baseline had a significant effect on treatment success in the univariable logistic regression analysis (*P *= 0.015; Table 3), with treatment success increasing from 12.5% for patients with FTMH width at baseline >600 µm to 28.4% for patients with FTMH width at baseline <600 µm. None of the other patient characteristics previously shown to be predictive for VMA resolution, including younger age, phakic lens status, or absence of ERM,^[14,33]^ showed a statistically significant association with treatment success (Table 3). As the two significant patient baseline characteristics are necessarily highly correlated, and additionally FTMH width at RPE at baseline was unavailable for the OASIS dataset, they were not used jointly in a multivariable logistic regression analysis.

### Case studies

Two patients are herein presented as case studies to exemplify real-world clinical findings with ocriplasmin use in patients with symptomatic VMA and FTMH.

#### Case 1

**Figure 1:**
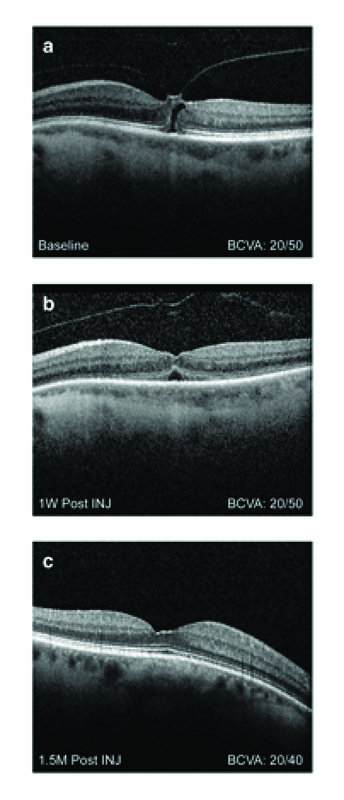
Case 1 Spectral domain optical coherence tomography of a 71-year-old female with VMA and a tractional macular hole in the left eye. (A) Baseline visit. No presence of ERM; BCVA 20/60. (B) One week post ocriplasmin injection. VMA resolved and macular hole closed, but increased presence of SRF; BCVA 20/50. (C). Seven weeks post treatment. Macular hole remains closed, no evidence of SRF; BCVA 20/40. BCVA, best-corrected visual acuity; ERM, epiretinal membrane; INJ, injection; M, month; SRF, subretinal fluid; W, week

A 71-year-old white woman had initial presentation of blurred central vision for four–six weeks and ghosting of letters while reading in the left eye. Medical and ocular history were noncontributory. Visual acuity was 20/60 at initial visit. SD-OCT revealed VMA with tractional macular hole of 300 μm, with no presence of ERM (Figure 1A). The left eye had phakic lens status. The patient opted for ocriplasmin treatment and received the intravitreal injection 18 days after initial visit. Visual acuity was 20/60 pre-injection.

**Figure 2:**
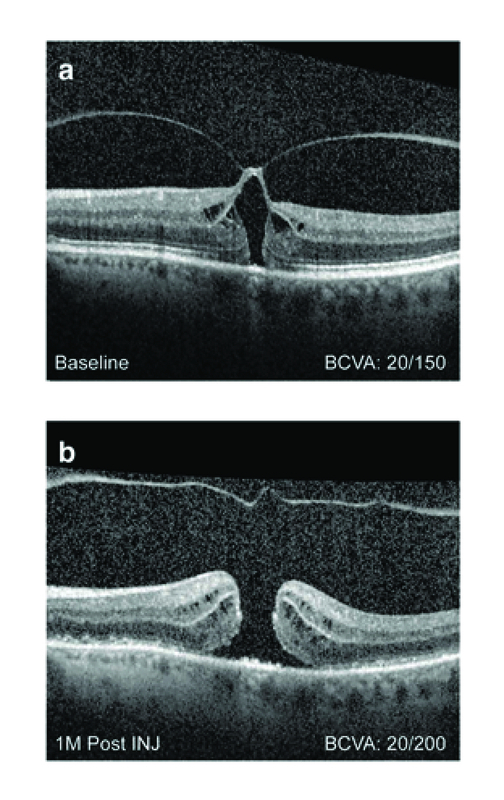
Case 2 Spectral domain optical coherence tomography of a 63-year-old female showing VMA with FTMH. (A) Baseline visit. No ERM or presence of SRF; BCVA 20/150. (B) One month post treatment. VMA released, but macular hole remained open, with the base enlarging to 1323 μm. BCVA decreased to 20/200. BCVA, best-corrected visual acuity; ERM, epiretinal membrane; INJ, injection; M, month; SRF, subretinal fluid

One week following the ocriplasmin injection, VMA resolved and the macular hole closed (Figure 1B). However, there was increased presence of SRF (Figure 1B). Visual acuity remained at 20/50. At seven weeks post-treatment, macular hole remained closed with no evidence of SRF (Figure 1C). Visual acuity improved to 20/40.

#### Case 2 

A 63-year-old White woman initially presented with symptoms of blurred central vision for two–three months in the left eye. Medical history included essential hypertension. Visual acuity was 20/150 at initial visit. Patient had phakic lens status in the left eye. Upon examination, SD-OCT showed FTMH with VMA, with no ERM or presence of SRF (Figure 2A). The size of the tractional macular hole size at baseline was 145 μm, minimum linear diameter (MLD). The patient opted for ocriplasmin treatment and received the intravitreal injection 14 days after the initial visit. Pre-injection visual acuity was 20/150.

One month following the treatment with ocriplasmin, the VMA released, but the macular hole remained open, enlarging to a size of 428 μm, MLD (Figure 2B). Visual acuity decreased to 20/200. The patient underwent standard macular hole repair via vitrectomy, internal limiting membrane peeling, and gas injection. The hole did not close and subsequent surgery including an internal limiting membrane patch and silicone oil was performed with macular hole closure. At the last examination, visual acuity was count fingers (CF) at 4 ft with a dense cataract and macular hole closure by OCT.

##  DISCUSSION

This study is the first to examine the baseline predictors of success for both VMA resolution and FTMH closure following ocriplasmin treatment. Our results show that FTMH ≤ 250 μm at baseline is significantly associated with VMA release by Day 28 and FTMH closure by Month 6 (*P* = 0.009), and may be the only positive baseline predictor for both pharmacological VMA release and nonsurgical FTMH closure, including previously identified predictors such as age, lens status, and absence of ERM.

Baseline factors associated with successful VMA release following ocriplasmin treatment have been widely studied following approval in 2012.^[14,15,16,17,18,19,20,21,22,23,24,25,26,27,28,29,30,31,32]^ A post hoc analysis of the phase 3 MIVI-TRUST trials revealed that baseline characteristics such as younger age, focal adhesions (VMA ≤ 1500 μm), phakic lens status, and absence of ERM promoted VMA resolution,^[14]^ and these characteristics have since been confirmed in multiple studies.^[15,16,17,18,19,20,21,22,23,24,25,26,27,28,29,30,31,32]^ These predictive characteristics were also shown to statistically favor VMA release (odds ratios 2.37–7.85) in a meta-analysis of 19 studies published in 2016.^[33]^

However, in our current analysis, most of these validated baseline factors were not shown to be predictive when analyzed for both VMA release and FTMH closure. The baseline factors of younger age, absence of ERM, and lens status did not reach statistical significance, with only FTMH size of ≤ 250 μm at baseline emerging as the only statistically significant factor favoring both VMA release and FTMH closure.

The fact that lens status was no longer significant in the multivariable model is due to the correlation between variables. The lens status of younger patients was more frequently phakic compared to older patients (93.6% vs 71.8%), and similarly, the lens status of patients without ERM at baseline was more frequently phakic compared to patients with ERM at baseline (83.0% vs 57.9%). For FTMH, the percentage of phakic lens status increases with increasing FTMH size, with 80.0% (60/75), 85.2% (46/54), and 100.0% (24/24) for the ≤250 µm, >250–400 µm, and >400 µm FTMH size categories, respectively, and with increasing FTMH width, with 85.0% (34/40) and 89.7% (35/39) for the ≤600 µm and >600 µm FTMH width at RPE categories, respectively.

Historically, whether FTMH at baseline serves as a predictive factor for successful VMA release has remained unclear. The presence of FTMH was initially identified as a predictive characteristic in the post hoc analysis of the MIVI-TRUST trials.^[14]^ Subsequently, Chatziralli et al performed a meta-analysis and did not conclude that the presence of FTMH was a predictive factor for VMA release.^[33]^ However, only 8 of the 19 analyzed studies assessed MH size as a predictive factor.^[13,15,17,23,24,28,30,32]^ Kuppermann (2015)^[39]^ reported the results of 10 retrospective studies which assessed the presence of FTMH on VMA resolution, including 4 studies not included in Chatziralli et al.^[40,41,42,43]^ Eight of these 10 studies^[19][23][31][32][40,41,42,43]^ showed that the subgroup of patients with a FTMH had higher VMA resolution rates than those without.^[39]^ These results were also consistent with the prospective OASIS trial.^[34]^ However, other studies have not shown greater rates of VMA resolution in patients with FTMH at baseline.^[28,29]^ Therefore, the value of FTMH as a predictive factor for VMA resolution needs to be further elucidated.

In our current analysis, the majority of patients failing to achieve both VMA resolution and FTMH closure were due to lack of macular hole closure. Whereas VMA resolution rates were 50% or higher from all studies in this patient population (i.e., those with symptomatic VMA and FTMH at baseline treated with ocriplasmin with at least one follow-up visit), FTMH closure rates for OASIS and ORBIT studies were lower than that of the original phase 3 MIVI-TRUST trials, albeit higher than the closure rates experienced in the control groups (15.4% and 10.6%, respectively). These results suggest that the known baseline factors predictive of VMA resolution, which were used as key inclusion criteria for the OASIS study, may be necessary but not sufficient to predict FTMH closure. Nevertheless, consistent with our findings, previous studies investigating FTMH closure rates following ocriplasmin treatment have repeatedly shown FTMH size at baseline to be the most consistent predictive factor, with a greater proportion of patients experiencing hole closure with an FTMH ≤ 250 μm compared to those with an FTMH > 250–400 μm.^[14,36,37,44]^ In contrast, the natural history of untreated FTMH has revealed that spontaneous closure rates are low, ranging from 3–11%.^[45,46,47,48,49]^ Although smaller holes have a comparatively better chance of spontaneous closure compared to larger ones, previous studies have shown that the majority of stage 2 macular holes (<400 μm) progress to stage 3 and beyond if left untreated.^[50,51,52,53]^

Whether VMA resolution is correlated with FTMH closure has also remained unclear. Recently, Feng et al demonstrated that successful VMA resolution was a statistically significant positive predictor for FTMH closure following ocriplasmin treatment (*P *= 0.042).^[37]^ This is consistent with our findings, which showed that patients with VMA resolution by Day 28 had a significantly higher rate of FTMH closure compared to those without VMA resolution. However, other analyses have not shown an association between VMA resolution and FTMH closure. In one study, 40% of patients required surgical closure for macular holes despite successful VMA resolution,^[54]^ suggesting that additional factors may impact FTMH closure.

Although our finding that VMA resolution showed a positive correlation with FTMH closure is notable, beyond initial hole size, baseline characteristics predictive of macular hole closure prior to treatment have remained elusive. For instance, our findings are consistent with previous analyses showing that, unlike for VMA resolution, absence of ERM did not significantly impact FTMH closure rates.^[35,36]^ Additional studies have suggested that other factors, such as macular hole architecture, may affect closure.^[55,56]^ Recently, Steel et al found that macular hole “width factor,” defined as the base diameter (BD) minus the MLD, was the most predictive factor of macular hole closure; holes having a BD close in size to the MLD were shown to have higher probability of closure compared to those with a wider base.^[56]^ A similar outcome is shown in Case 2, where despite VMA resolution, the macular hole widens at the base with the edge elevated by a cuff of SRF. This is consistent with previous cases showing failure of FTMH closure due to base enlargement following ocriplasmin treatment and subsequent VMA resolution.^[37,57]^ SRF did not have a statistically significant predictive value in our analysis; however, the number of patients showing successful VMA resolution and FTMH closure with SRF were strikingly different between the MIVI-TRUST and OASIS vs ORBIT studies (SRF present: 87% [20/23], 100% [8/8], and 0% [0/30], respectively), perhaps owing in part to differences in SRF measurement protocols at study enrollment and therefore limiting interpretation. In Case 1, presence of SRF did not impact VMA resolution or FTMH closure, although visual acuity improved following SRF resolution.

When selecting a treatment option for patients with VMA and FTMH, the risks and benefits of ocriplasmin versus vitrectomy should be carefully considered. For these patients, vitrectomy is considered the standard of care, with macular hole closure rates reported for 87.5% of patients in a meta-analysis.^[33,58,59]^ However, persistence of a macular hole after vitrectomy remains one of the major complications of this type of surgery, with approximately one in eight macular holes failing to close.^[58]^ A persistent macular hole typically increases in diameter, with an accompanying loss of visual acuity, and studies have shown lower treatment success for subsequent surgery.^[55,58]^ Additional complications of vitrectomy include cataract formation, retinal detachment, and hemorrhage.^[33,59,60,61,62,63,64]^ In addition, based on the OASIS trial, patients who underwent vitrectomy experienced retinal tear and retinal detachment more often than patients receiving ocriplasmin. Most adverse events in the ocriplasmin group were transient in nature, had a short onset time, and were mild to moderate in severity.^[34]^

Strengths of the current analysis include a robust and homogeneous patient sample pooled from multiple clinical trials, utilizing the same ocriplasmin treatment regimen. Limitations include the post hoc nature of the analysis, which was not prespecified in the clinical trials, as well as the lack of availability of certain baseline ocular characteristics in all trials.

Since the pivotal clinical trials, continued study and analysis has been undertaken to more fully understand the efficacy and safety of ocriplasmin, including the baseline characteristics predictive of VMA resolution and FTMH closure. These results suggest that patients presenting with symptomatic VMA and FTMH ≤ 250 μm may be ideal candidates for ocriplasmin treatment.

##  Financial Support and Sponsorship

This study was funded by Oxurion. All authors, including those affiliated with the study sponsor, were involved in the design of the study, interpretation of the data, writing of the manuscript, and the decision to submit the manuscript for publication.

##  Conflicts of Interest

The authors declare the following potential conflicts of interest with respect to the research, authorship, and/or publication of this article:

BJ: Consultant for Oxurion; PW: Consultant for Oxurion; TR: Employee of Oxurion; LD: Consultant for Oxurion; JM: Consultant for Oxurion.

*Research involving human participants and informed consent*

This manuscript represents a retrospective analysis of four prospective phase 3/4 clinical trials.

Full details of adherence to ethics practices have been previously published ^[13,34][38]^.

##  Author Contribution

All authors contributed to the conception or design of the work, analysis and interpretation of the data, critical revision of the manuscript, and approval of the final version to be published.
